# Suicidal ideation and associated factors among people living with HIV/AIDS in Ethiopia: a systematic review and meta-analysis

**DOI:** 10.3389/fpsyt.2024.1361304

**Published:** 2024-09-10

**Authors:** Amanuel Yosef Gebrekidan, Afework Alemu Lombebo, Amelework Gonfa Efa, Gedion Asnake Azeze, Gizachew Ambaw Kassie, Kirubel Eshetu Haile, Yordanos Sisay Asgedom, Beshada Zerfu Woldegeorgis, Tadesse Asmamaw Dejenie

**Affiliations:** ^1^ School of Public Health, College of Health Sciences and Medicine, Wolaita Sodo University, Wolaita Sodo, Ethiopia; ^2^ School of Medicine, College of Health Sciences and Medicine, Wolaita Sodo University, Wolaita Sodo, Ethiopia; ^3^ School of Midwifery, College of Medicine and Health Sciences, Hawassa University, Hawassa, Ethiopia; ^4^ School of Nursing, College of Health Sciences and Medicine, Wolaita Sodo University, Wolaita Sodo, Ethiopia; ^5^ Department of Internal Medicine, College of Health Sciences and Medicine, Wolaita Sodo University, Wolaita Sodo, Ethiopia; ^6^ Department of Medical Biochemistry, College of Medicine and Health Sciences, University of Gondar, Gondar, Ethiopia

**Keywords:** HIV, AIDS, Ethiopia, suicide, suicidal ideation

## Abstract

**Background:**

Suicide is one of the main causes of mortality in the world, accounting for more fatalities than homicide, war, human immunodeficiency virus (HIV)/acquired immunodeficiency syndrome (AIDS), breast cancer, and malaria. Significantly, the biggest risk factors for suicide in the general population are having already attempted suicide and suicidal ideation. Despite the availability of studies on suicidal ideation among people living with HIV/AIDS (PLWHA) in Ethiopia, the results are inconsistent. Thus, a systematic review and meta-analysis was conducted to estimate the pooled prevalence of suicidal ideation among people living with HIV/AIDS.

**Methods:**

A systematic review and meta-analysis was conducted in accordance with the Preferred Reporting Items for Systematic Reviews and Meta-Analyses (PRISMA) guideline. To find papers published in the English language before 20 May 2023, the electronic databases of Medline, Science Direct, Excerpta Medica Database, Cochrane Library, African Journals Online (AJOL), and Google Scholar were searched. The DerSimonian and Laird method for random effects models was used to estimate the pooled prevalence of suicidal ideation with a 95% confidence interval in STATA V.14.0 statistical software. To test for heterogeneity between studies and publication bias, respectively, forest plots and funnel plots were used. Additionally, leave-one-out sensitivity was conducted.

**Results:**

A total of nine studies with 3,411 study participants were included in this systematic review and meta-analysis. The pooled prevalence of suicidal ideation among PLWHA was 20.55% (95% CI 14.76, 26.33). Being female (Odds ratio (OR) = 4.27, 95% CI = 2.29, 7.97), living alone (OR = 5.02, 95% CI = 2.15, 11.64), poor social support (OR = 3.80, 95% CI = 2.56, 5.65), perceived stigma (OR = 3.50, 95% CI = 1.55, 7.87), depression (OR = 5.08, 95% CI = 2.55, 11.48), undisclosed HIV status (OR = 4.8, 95% CI = 2.10, 10.93), and World Health Organization HIV clinical stages of III or IV (OR = 4.40, 95% CI = 2.95, 6.58) were significantly associated with suicidal ideation.

**Conclusion:**

Suicidal ideation among PLWHA is high in Ethiopia. Therefore, emphasis should be given to psychiatric assessment and interventions with a special focus on individuals having the associated factors.

**Systematic Review Registration:**

PROSPERO (CRD42023429613).

## Introduction

Suicide is often given low priority by governments and policy-makers even though many of these deaths are preventable and every year many more people attempt suicide than there are suicides (1, 2). Suicide is one of the main causes of mortality in the world, accounting for more fatalities than homicide, war, human immunodeficiency virus/acquired immunodeficiency syndrome (HIV/AIDS), breast cancer, and malaria (1, 3). By 2020, the annual worldwide suicide rate was predicted to increase to 11.4 per 100,000 people, accounting for roughly 2.4% of the world’s illness burden, with one suicide-related death occurring every 20 seconds. Furthermore, people living with HIV/AIDS (PLWHA) have 9.6 times higher mortality rates due to suicide than the overall population (4, 5).

Each year, more than 700,000 people die by suicide. Suicide is the fourth-leading cause of death among people aged 15 to 29 (6). Low- and middle-income nations account for more than three-fourths of all suicides worldwide (6). The overall pooled prevalence of suicidal ideation according to the Global School-based Student Health Survey (GSHS) of adolescents aged 12–17 years between 2003 and 2015 from 82 low, middle, and high income countries was 14% (7). A systematic review and meta-analysis conducted in Ethiopia found that the pooled prevalence of suicidal ideation in the general population was 9% (8).

Over 0.5 percent of the global population is infected with HIV. Every day, approximately 5,000 new infections arise (9). Worldwide, 39 million people were living with HIV in 2022 of which 1.3 million people were newly infected with HIV in 2022. In total, 20.8 million people were living with HIV in 2022 in Eastern Africa and South Africa, accounting for the majority of the global burden (10). According to the 2021 data from the Joint United Nations Programme on HIV/AIDS (UNAIDS), 610,100 people live with HIV in Ethiopia and 12000 individuals died due to HIV/AIDS-related illness (11). Adult HIV prevalence in Ethiopia was found to be 3.0%; however, the prevalence varied significantly by administrative region, from 0.8% in Somalia to 5.7% in Gambela (11, 12). These figures reflect the high prevalence of HIV/AIDS worldwide, including in Ethiopia, demonstrating a considerably high number of susceptible individuals to suicide compared to the general population.

Compared to the general population, the prevalence of mental health issues including suicide is higher among PLWHA and HIV-vulnerable individuals. Psychiatric problems such as depression and suicidality are common among HIV-positive people with an estimated global prevalence of 31% with a higher prevalence of depression being recorded in underdeveloped and developing countries compared to developed countries (13–15).

Depressive disorders are one of the top direct causes of disease burden globally, and it has been shown that major depressive disorders also increase the burden related to suicide (16). In contrast to the overall population, at every stage of the HIV care continuum, PLWHA who have mental health issues are more likely to experience adverse health consequences (15). A systematic review and meta-analysis of the pooled prevalence estimates found the pooled prevalence of suicidal ideation, attempted suicide, and suicide deaths among PLWHA to be 22.3%, 9.6%, and 1.7%, respectively (17). Furthermore, a systematic review conducted on the global prevalence of lifetime suicidal ideation among PLWHA found the pooled prevalence to be 22.4%, while in Africa it was found to be 21.7% (18, 19). Different studies conducted on the prevalence of suicidal ideation among PLWHA in Ethiopia show a prevalence range of 8.2%-33.6% (20, 21).

Among the factors associated with suicidal ideation, HIV status non-disclosure, polygamous family, physical and emotional abuse, primary school education, and a decline in academic performance were also among the factors associated with suicidal ideation in PLWHA in Africa (19). Studies conducted in Ethiopia have mentioned socio-demographic and economic factors such as female sex, marital status, social support, living alone, and monthly income as factors associated with suicidal ideation in PLWHA (3, 21–26).

Clinical factors such as CD4 level, WHO clinical stage, not being on highly active antiretroviral therapy (HAART), low body mass index, undisclosed HIV status, and having a comorbid opportunistic infection were mentioned as associated factors of suicidal ideation in PLWHA (3, 20, 21, 23–26).

Despite the availability of studies on suicidal ideation among PLWHA in Ethiopia, the results are inconsistent. The prevalence of suicidal ideation among PLWHA ranged from a low of 8.2% (20) to a high of 33.6% (21). Therefore, by gathering information from the current scientific literature, we conducted a systematic review and meta-analysis to estimate the pooled prevalence of suicidal ideation among PLWHA in Ethiopia.

## Method

### Study design

A systematic review and meta-analysis of observational studies was conducted on suicidal ideation and associated factors among people living with HIV/AIDS in Ethiopia. All studies on suicidal ideation and associated factors among PLWHA which were published up to 20^th^ May 2023 were retrieved using the Preferred Reporting Items for Systematic Reviews and Meta-Analyses (PRISMA) guidelines (27) (**Supplementary File 1**). The systematic review and meta-analysis protocol for this study was registered on PROSPERO under reference number CRD42023429613.

### Search strategy

We carried out a systematic search in the electronic databases of Medline, Science Direct, Excerpta Medica Database, Cochrane Library, AJOL, and Google Scholar to find all pertinent observational studies on suicidal ideation and associated factors among PLWHA in Ethiopia up to 20^th^ May 2023. Using EndNote Ref Manager version 20, articles were downloaded, organized, and cited. A manual search was also conducted to find additional potentially relevant research using the reference lists of the papers that were retrieved. Only studies conducted in the English language were included. The search was carried out using the following keywords: (suicidal ideation OR suicide OR suicidal attempt OR suicidal behavior OR suicidal thought OR suicidal plan) AND (people OR individuals OR patients OR male OR Female OR adults OR mothers)) AND (HIV OR Human Immunodeficiency Virus OR AIDS OR acquired immunodeficiency syndrome)) OR (associated factors OR risk factor OR risk factors OR risk OR determinant factors OR determinants factors)) AND (epidemiology OR epidemiology OR prevalence OR (incidence)) AND (Ethiopia OR Northern Ethiopia OR Southern Ethiopia OR Eastern Ethiopia OR Western Ethiopia OR Central Ethiopia)) (**Supplementary File 2**). We considered studies that examined the prevalence of suicidal ideation among people living with HIV/AIDS in Ethiopia. Articles that featured human subjects, were observational in nature, and had been published in English were included. Using MeSH (Medical Subject Headings) and Boolean operators, databases were searched.

### Eligibility criteria

Studies that fulfilled the following criteria were included: i) Study period: studies conducted or published until 20^th^ May 2023; ii) Study type: observational studies; iii) Population: Studies conducted on PLWHA; iv) Outcome; suicidal ideation (Proportion); v) Place of study: studies conducted in Ethiopia; and vi) Studies published in the English language. Review articles were excluded.

CoCoPop: Condition: suicidal ideation; Context: Ethiopia; Population: people living with HIV/AIDS.

### Study selection and extraction

**Figure 1:**
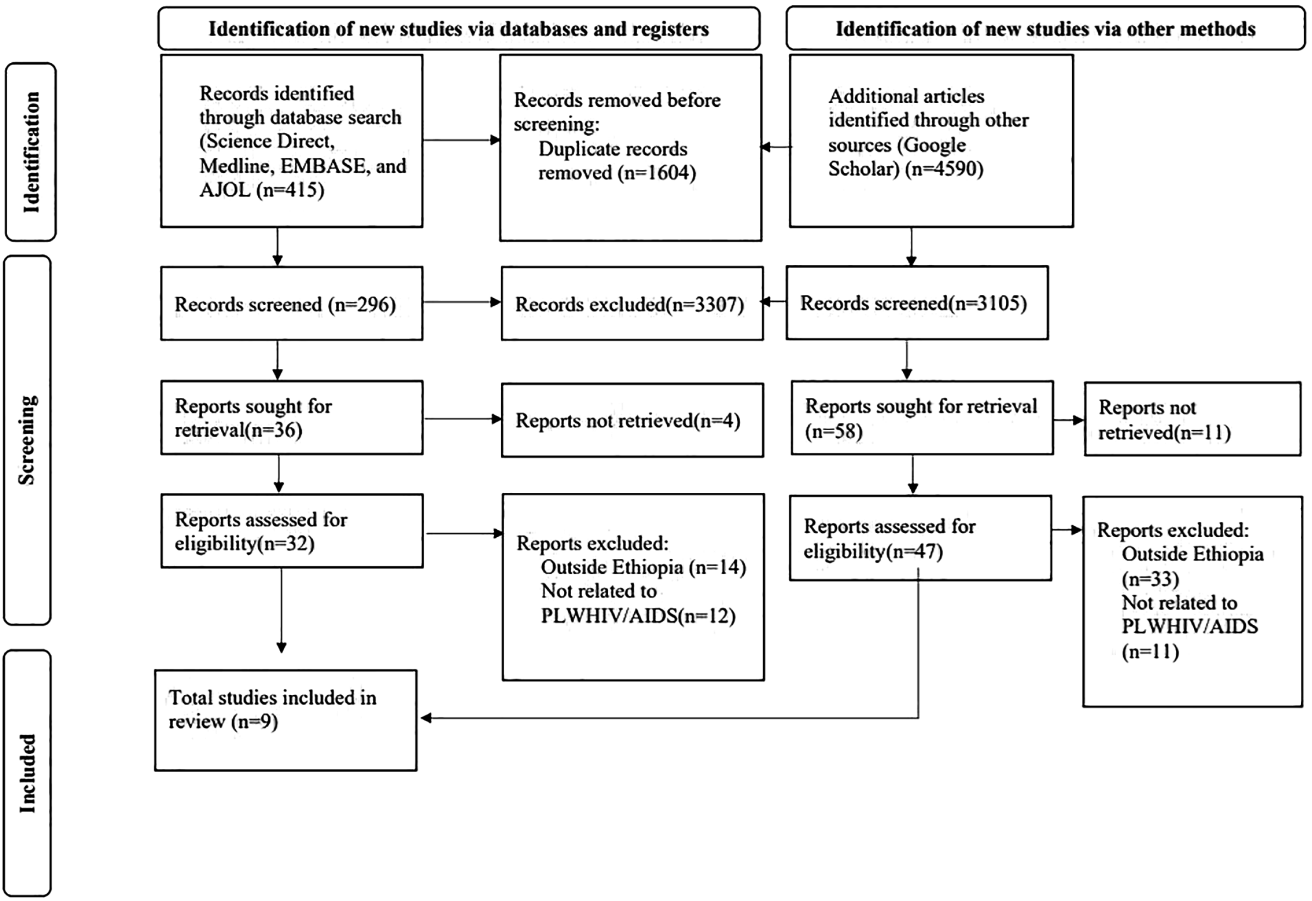
PRISMA flow diagram of the selection process of studies on suicidal ideation among PLWHA in Ethiopia.

The retrieved studies were imported into EndNote (Version 20, for Windows, Thomson Reuters, Philadelphia, PA, USA), where 1,604 duplicate studies were removed. All of the papers were evaluated by three independent reviewers (AYG, AAL, and YSA) for eligibility; the abstract and title were checked first, followed by the entire text. Four investigators (AGE, TAD, GAA, and KEH) independently used a consistent data extraction format created in Microsoft Excel to extract the data. Prior to the extraction procedure, the three independent researchers were blinded to any study data. First author’s name, year of publication, region, study area, sample size overall, response rate, prevalence of suicidal ideation, and quality rating were among the variables extracted (**Figure 1**) (**Table 1**).

**Table 1:** Characteristics of the nine studies included in the systematic review and meta-analysis of suicidal ideation among PLWHA in Ethiopia.

S. N	Author's Name	Publication year	Region	Study design	Population	Sample size	Response rate (%)	Prevalence (%)
1	Selamawit et al (30)	2015	Amhara	Cross-sectional	Adults	422	97.6	22.1
2	Huluagresh et al (21)	2016	Amhara	Cross-sectional	Adults	393	100	33.6
3	Etsay et al (24)	2017	Addis Ababa	Cross-sectional	Adults	423	98.6	22.5
4	Mesele et al (3)	2019	Addis Ababa	Cross-sectional	Youth	423	97.6	27.1
5	Kefyalew et al (22)	2021	Amhara	Cross-sectional	Adults	348	93.7	16
6	Leul et al (20)	2021	Amhara	Cross-sectional	Pregnant	414	98.1	8.2
7	Koku et al (25)	2022	Amhara	Cross-sectional	Adults	395	100	9.4
8	Gebreslassie et al (23)	2022	Harari	Cross-sectional	Adults	423	97.4	24.3
9	Mikiyas et al (26)	2023	Addis Ababa	Cross-sectional	Addis Ababa	237	100	22.8

### Quality assessment

Following the full-text review, three authors (AYG, GAK, and BZW) used the Newcastle-Ottawa Quality Assessment Scale (adapted for cross-sectional studies) (28) to evaluate the article’s quality. Any disagreements were settled by discussion and consensus. We used the following items for assessment of the included studies: 1) Representativeness of the sample; 2) Sample size; 3) Non-response rate; 4) Ascertainment of the exposure (risk factor); 5) The subjects in different outcome groups are comparable, based on the study design; 6) Assessment of the outcome; 7) Statistical test. Articles with a quality assessment checklist criteria score of ≥5 were considered studies with low risk and these studies were included in the systematic review and meta-analysis (**Table 2**).

**Table 2:** Quality ratings of studies included in the systematic review and meta-analysis of suicidal ideation among PLWHA in Ethiopia.

	Study	Selection				Comparability	Outcome		
S.N		Representativeness of the sample	Sample size	Non respondents	Ascertainment of the exposure (maximum score=2)	The subjects in different outcome groups are comparable based on the study design or analysis. Confounding factors are controlled (maximum score=2)	Assessment of the outcome(maximum score=2)	Statistical test	Total (10)
1	Selamawit et al (30)	1	1	1	1	1	1	0	6
2	Huluagresh et al (21)	1	1	1	1	1	2	0	7
3	Etsay et al (24)	1	0	1	1	1	2	1	7
4	Mesele et al (3)	1	1	1	1	1	2	1	8
5	Kefyalew et al (22)	1	1	1	1	1	2	1	8
6	Leul et al (20)	1	1	1	1	1	2	1	8
7	Koku et al (25)	1	1	1	1	1	2	1	8
8	Gebreslassie et al (23)	1	1	1	1	1	2	1	8
9	Mikiyas et al (26)	1	1	1	1	1	2	1	8

### Statistical analysis

STATA 14.0 software (StataCorp, College Station, Texas, USA) was used to analyze the data. We used the DerSimonian and Laird method for random effects models to calculate the pooled prevalence of suicidal ideation among PLWHA in Ethiopia (29). The I2 statistical test was conducted to examine study heterogeneity. I^2^ values of 0%, 25%, 50%, and 75% were interpreted as correspondingly denoting no, low, medium, and high heterogeneity. In order to estimate pooled prevalence with 95% confidence intervals (CI), a meta-analysis with a random effects model was done due to the considerable heterogeneity that was discovered between the studies (p<0.01, I^2^ >95.3%). The meta-analysis’s findings were displayed using a forest plot. Egger’s test was performed to determine whether publication bias existed, and any possible bias was also identified visually by examining the funnel plot. To pinpoint the key studies that had the most significant influence on the between-study heterogeneity, a leave-one-out sensitivity analysis was also carried out. By omitting each study individually, the analysis was conducted to determine the impact of each study on the pooled estimate of suicidal ideation among PLWHA in Ethiopia. The input variables needed by the cells of the two-by-two tables for factors related to suicidal ideation are binary data, or “determinants of suicidal ideation,” i.e., the proportion of people in each study’s exposed and non-exposed groups who had and did not have suicidal ideations. The odds ratio (OR), which was computed based on the binary results of the included main studies, was used to evaluate all relevant factors related to suicidal thoughts. The pooled odds ratio was calculated using a random effect meta-analysis, and a 95% confidence interval was employed. The effect magnitude and 95% confidence interval results were shown as forest plots.

## Result

A total of 5,005 articles were retrieved that had been published before 20^th^ May 2023 using the electronic databases. In total, 1,604 articles were deleted due to duplication. Of the remaining 3,401 articles, 3,307 were removed by title and abstract, while 94 were read in full and assessed for eligibility. Finally, nine studies with a total of 3,411 study participants met the eligibility criteria and were included in the meta-analysis (**Figure 1**) (**Table 1**).

### Characteristics of included studies

Of the nine included studies, five were conducted in the Amhara region (20–22, 25, 30), three were conducted in Addis Ababa (3, 24, 26), and the remaining study was conducted in the Harari region (23). The highest prevalence (33.6%) of suicidal ideation among PLWHA in Ethiopia was reported by a study conducted in the Amhara region (21), while the lowest prevalence (8.2%) was documented in a study conducted in the Amhara region including pregnant women with HIV/AIDS (20). Eight studies included adult PLWHA with ages greater than or equal to 18 (21–26, 30), while one study included youths (3) and one other study included pregnant women living with HIV/AIDS (20). Eight studies used the Composite International Diagnostic Interview while one study used the WHO self-reporting questionnaire (SRQ-20) (30).

### Pooled prevalence of suicidal ideation among PLWHA in Ethiopia

**Figure 2:**
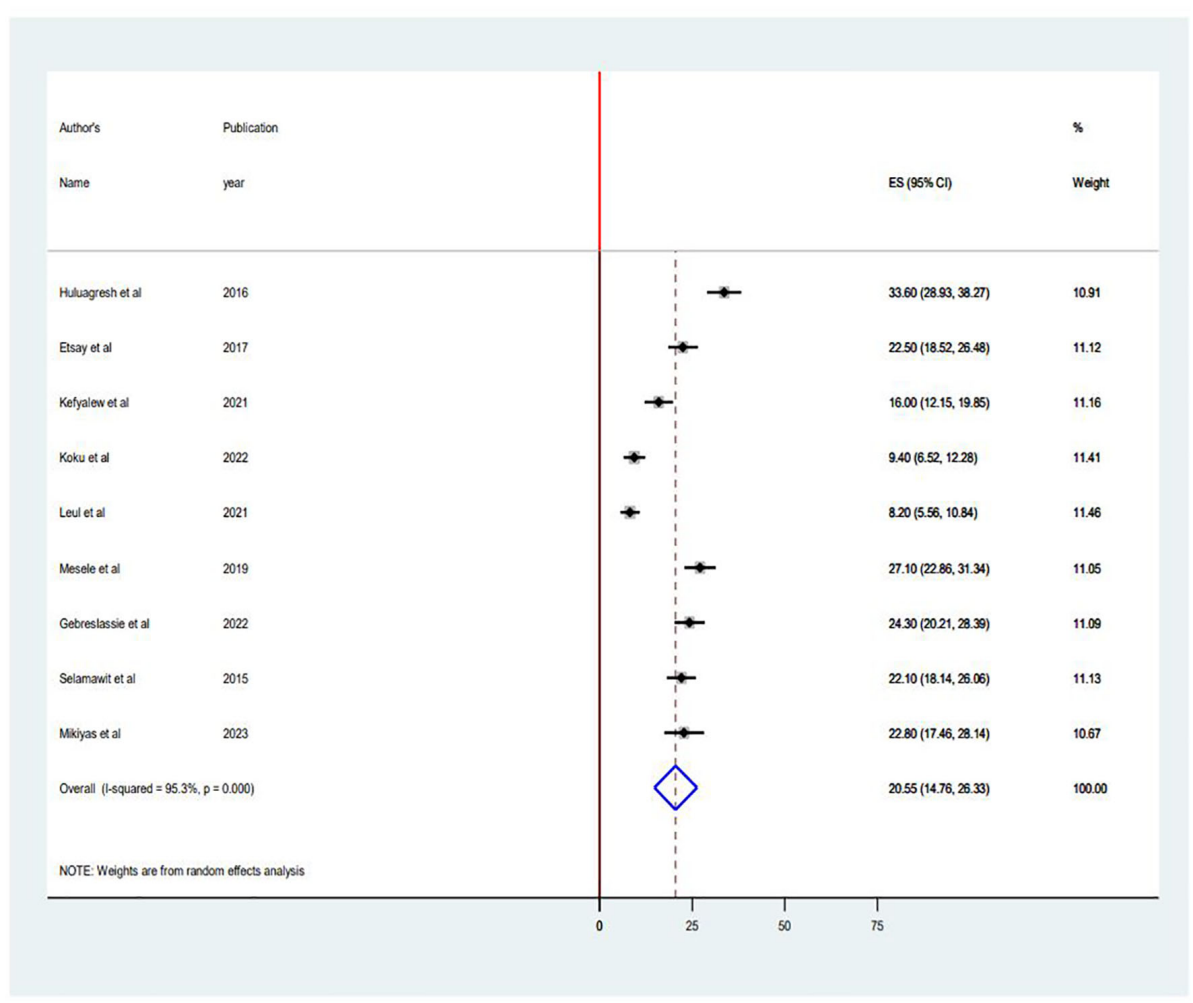
Forest plot of the pooled prevalence in the systematic review and meta-analysis of suicidal ideation among PLWHA in Ethiopia.

The pooled prevalence of suicidal ideation among PLWHA in Ethiopia was 20.55% (95% CI 14.76, 26.33). The forest plot below shows a statistically significant heterogeneity (I^2^ = 95.3%; p < 0.001) (**Figure 2**). Therefore, a random effects model was used to estimate the pooled prevalence of suicidal ideation among PLWHA in Ethiopia.

### Subgroup analysis

**Figure 3:**
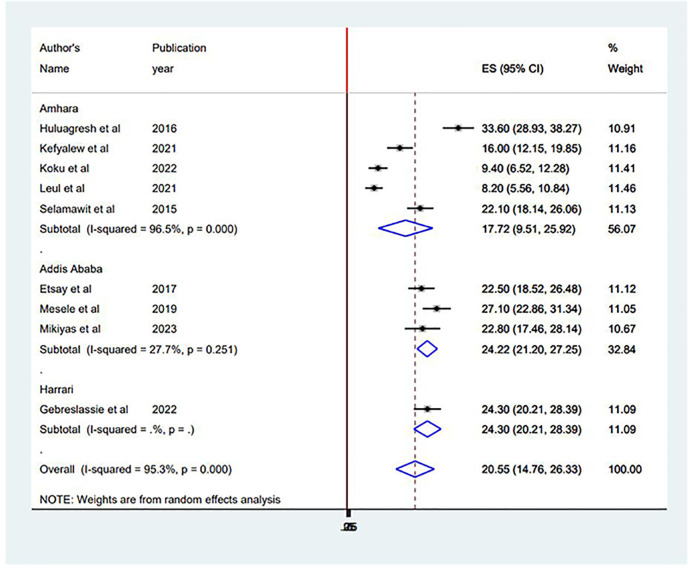
Subgroup analysis based on study area (region) in the systematic review and meta-analysis of suicidal ideation among PLWHA in Ethiopia.

A subgroup analysis was undertaken to determine the sources of heterogeneity among the studies due to the significantly high heterogeneity. Subgroup analysis was conducted based on the study area (regions) (**Figure 3**) and publication year to identify the possible sources of heterogeneity. Regarding the sub-group analysis by region, the highest pooled prevalence of suicidal ideation was reported in Addis Ababa with 24.23% (95% CI 21.69, 26.78), and the lowest was documented in the Amhara region with 14.37% (95% CI 12.87, 15.88). As for publication year, the highest pooled prevalence of suicidal ideation was reported in studies conducted before 2020 with 26.21% (95% CI 21.2, 31.22), and the lowest was documented in studies conducted after 2020 with 15.93% (95% CI 9.62, 22.23).

### Publication bias

**Figure 4:**
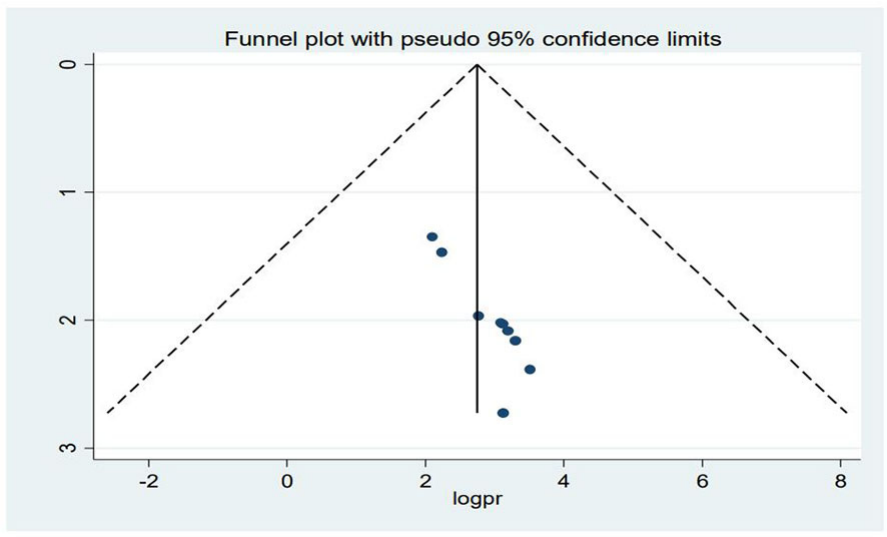
Funnel plot of publication bias in the systematic review and meta-analysis of suicidal ideation among PLWHA in Ethiopia.

The graphical distribution of the funnel plot shows evidence of asymmetry (**Figure 4**). The result of the Egger’s test was also statistically significant with a coefficient = 1.16, 95% CI (0.74, 1.58), and a p-value of <0.001. As a result, we used a trim-and-fill analysis to estimate the number of potentially missed studies by filling in a potential four studies and adjusting for the overall effect estimate. Using the trim and fill analysis, the overall polled estimate became 14.36%, 95% CI (8.36, 20.35).

### Sensitivity analysis

#### Leave-one-out sensitivity analysis

By excluding each included study individually, the leave-one-out sensitivity analysis was used to assess the impact of each study on the pooled prevalence of suicidal ideation among PLWHA in Ethiopia. The findings revealed that the studies that were removed had no discernible impact on the pooled prevalence of suicidal ideation among PLWHA in Ethiopia.

### Factors associated with suicidal ideation among PLWHA in Ethiopia

To determine the factors associated with suicidal ideation among PLWHA in Ethiopia, variables such as sex, marital status, living status, social support, social stigma, depression, HIV status disclosure, CD_4_ level, and WHO clinical stages were extracted from the included studies. Out of those variables, seven variables were identified as factors associated with suicidal ideation among PLWHA: sex, living status, social support, perceived stigma, depression, HIV status disclosure, and WHO clinical stages (**Table 3**).

**Table 3:** Factors associated with suicidal ideation among PLWHA in Ethiopia.

Determinants (ref no.)	Number of studies	Sample size	OR (95% CI)	P-value	I^2^ (%)	Heterogeneity test (p-value)
Sex (3, 21)	2	816	4.27(2.29-7.97)	<0.001	65.4	0.089
Living status (22, 23, 26)	3	1008	5.0(2.15-11.64)	<0.001	80.7%	0.006
Social support (21, 25)	2	788	3.8(2.56-5.65)	<0.001	0%	0.539
Perceived stigma (3, 24)	2	846	3.5(1.55-7.87)	0.002	80.6%	0.023
Depression (3, 21, 24, 25)	4	1634	5.08(2.55-11.48)	<0.001	88.1%	<0.001
HIV status disclosure (20, 26)	2	651	4.8(2.10-10.93)	<0.001	59%	0.119
WHO clinical stage (3, 24)	2	846	4.4(2.95,6.58)	<0.001	0%	0.784

In this systematic review and meta-analysis, women were 4.27 times more likely to have suicidal ideation than men (OR = 4.27, 95% CI = 2.29, 7.97). The odds of suicidal ideation were five times higher in individuals living alone compared to those who lived with others (OR = 5.02, 95% CI = 2.15, 11.64).

PLWHA who reported having poor social support were 3.8 times more likely to have suicidal ideation than those who reported good social support (OR = 3.80, 95% CI = 2.56, 5.65).

PLWHA who reported perceived stigma were 3.5 times more likely to have suicidal ideation compared to those who did not report perceived stigma (OR = 3.50, 95% CI = 1.55, 7.87).

The odds of suicidal ideation were 5.08 times higher in individuals with depression compared to those who had no depression (OR = 5.08, 95% CI = 2.55, 11.48).

Individuals with undisclosed HIV status were 4.8 times more likely to have suicidal ideation than individuals with a disclosed HIV status (OR = 4.8, 95% CI = 2.10, 10.93). Additionally, PLWHA who were classified stages III or IV based on the WHO clinical staging had 4.4 times higher odds of having suicidal ideation than those who were classified as stage I (OR = 4.40, 95% CI = 2.95, 6.58).

## Discussion

This systematic review and meta-analysis aimed to estimate the pooled prevalence of suicidal ideation among PLWHA in Ethiopia and found that the pooled prevalence of suicidal ideation among PLWHA in Ethiopia was 20.55% (95% CI 14.76, 26.33). This finding was in line with results from a systematic review and meta-analysis on the global prevalence of suicidal ideation, suicide attempts, and suicide planning, which reported a pooled prevalence of suicidal ideation of 20.9% (18). The finding of this systematic review and meta-analysis was also similar to the result from a systematic review and meta-analysis of the global prevalence of suicidal ideation and suicide attempts among young PLWHA with the pooled prevalence of lifetime suicidal ideation being reported as 24.38% (31) and the pooled prevalence of suicidal ideation, suicide attempts, and their associated factors among HIV/AIDS patients in Africa was 21.7% (19). A possible explanation might be due to the fact that more than 75% of the included studies were from low- and middle-income countries and specifically from sub-Saharan Africa, including studies from Ethiopia, with most of the study areas having similar socio-demographic and service-related factors.

However, the finding from this study was lower than a systematic review and meta-analysis conducted on the prevalence of suicide ideation among HIV/AIDS patients in China with a pooled prevalence of 30.6% (32). This might be due to the fact that half of the studies that were included in the systematic review and meta-analysis in China were conducted before 2014, as later studies may have been impacted by advancements in HIV/AIDS services.

However, the finding of this systematic review and meta-analysis was also higher than the pooled prevalence of suicidal ideation in the general population of Ethiopia at 9% (8). This difference can possibly be explained by the higher prevalence of depression and other mental illnesses in PLWHA compared to the general population (15).

The result of this systematic review and meta-analysis was also higher than a systematic review and meta-analysis of the global prevalence of suicide in PLWHA which reported a pooled prevalence of suicidal ideation of 0.909% (33). A possible explanation for this discrepancy might be the inclusion of more studies from middle- and high-income countries with better sociodemographic, economic, and health service characteristics.

The sub-group analysis based on publication year showed that there was a higher pooled prevalence of suicidal ideation, 26.21% (95% CI 21.20, 31.22), in the studies conducted before 2020 compared to the pooled prevalence of suicidal ideation in studies conducted after 2021, 15.93% (95% CI 9.62,22.23). This can be explained by improvements related to HIV services such as the provision of ART for all PLWHA without considering the WHO clinical staging.

The finding of this systematic review and meta-analysis found that women are more likely to have suicidal ideation compared to men. This finding is supported by a study conducted in Benin which stated that women living with HIV/AIDS are more likely to have suicidal ideation than their male counterparts (34). The finding is also supported by a study conducted in a Jamaican youth population (35) and a global systematic review and meta-analysis among PLWHA (36). This could be because women have a higher prevalence of depression and are less likely to express experiences that are stressful (8, 37).

This study found that the odds of suicidal ideation were higher in individuals living alone compared to those who lived with others. This finding is similar to a study conducted in the United States of America (USA) (38) in which loneliness was associated with suicidal ideation. A possible explanation might be the association of loneliness with poor physical and social support and a sense of isolation which in turn can lead to suicidality.

Additionally, this study showed that PLWHA who had poor social support were more likely to have suicidal ideation than those with good social support. This finding is similar to studies conducted on the relation of social support with suicidal ideation in China (39, 40). This might be due to social support helping PLWHA avoid suicidal thoughts by lowering HIV-related stress levels. Adequate social support may help patients handle stressful situations, shielding them from psychological suffering and thus lowering their chance of developing suicidal thoughts (39).

In this systematic review and meta-analysis, PLWHA with perceived stigma were more likely to have suicidal ideation compared to those who did not have perceived stigma. This result is supported by studies in Iran and China and another systematic review and meta-analysis (41–43). This might be due to self-imposed social isolation and a desire to avoid feeling guilty or judged because of having HIV. As a result, HIV stigma may have a negative impact on social networks and relationships, leading to depression and suicidal ideation (44, 45).

The odds of suicidal ideation were also higher in individuals with depression compared with those who did not have depression. This finding is in line with a systematic review and meta-analysis of suicidal ideation among PLWHA in Africa and a study in China (19, 46). This could be a result of the direct social effects of depression, such as social withdrawal, pessimism, and worthlessness, which can lead to suicidal ideation (19).

Individuals with an undisclosed HIV status had a higher likelihood of having suicidal ideation than individuals with a disclosed HIV status. This finding is also similar to a study conducted in West Africa and the USA (47). This is possibly due to individuals fearing that disclosure of their HIV status may lead to stigma and discrimination.

This systematic review and meta-analysis also found that PLWHA who are classified as stage III or IV based on the WHO clinical staging had higher odds of having suicidal ideation than those who were stage I. This finding is also supported by a systematic review and meta-analysis conducted in Africa (19). This can be explained by the presence of opportunistic infection with an associated decrease in quality of life.

## Limitations of the study

The presence of significant heterogeneity along with the small number of studies conducted in the study population may affect the generalizability of the study. The included studies were from two regions and one administrative city, further affecting the study’s generalizability to the whole country. The included studies are all cross-sectional. As a result, cause-and-effect relationships cannot be established between the factors and the outcome variable. Additionally, there is a possibility of social desirability bias among the study participants in the included studies due to the use of interviewer-administered questionnaires (3, 20, 24). Further, social desirability bias and recall bias may underestimate the real prevalence of suicidal ideation (3, 20, 22, 24). The use of clinically healthy respondents may also lower the real prevalence of suicidal ideation (25).

## Conclusion and recommendation

This systematic review and meta-analysis found that suicidal ideation among PLWHA is high in Ethiopia with one in five individuals living with HIV/AIDS reporting suicidal ideation. Sex, living alone, poor social support, perceived stigma, depression, HIV status disclosure, and WHO clinical stages III and IV were found to be factors associated with suicidal ideation. Therefore, psychiatric assessment and interventions should be routinely conducted with a special focus on individuals with these associated factors.

## Data Availability

The original contributions presented in the study are included in the article/**Supplementary Material**, further inquiries can be directed to the corresponding author.
