# Supplementary material for: Suicidal ideation and associated factors among people living with HIV/AIDS in Ethiopia: a systematic review and meta-analysis
Source: Front Psychiatry. 2024 Sep 10;15:1361304. doi: 10.3389/fpsyt.2024.1361304 (PMC11420569; doi:10.3389/fpsyt.2024.1361304)
Supplement: Supplementary file 2 [file Table2.docx]

**S2File: Search strategy for Suicidal ideation and associated factors among people living with HIV/AIDS in Ethiopia: A**

**systematic review and meta-analysis**

| **Database** | **Example of searching strategy** | **Number of studies** |
| --- | --- | --- |
| (Science Direct, Medline, Cochrane Library , Excerpta Medica, Scopus and AJOL) | (("Suicidal ideation*") OR (“suicide”) OR (suicidal attempt) OR (“suicidal behavior”) OR (“suicidal thought”) OR (“suicidal plan”))"[All Fields]) AND (("people”) OR (“individuals”) OR (“patients”) OR (“male”) OR (“female”) OR (“adults”) OR (“mothers”))*"[All Fields])) AND ((HIV) OR (Human Immunodeficiency Virus) OR (AIDS) OR (acquired immunodeficiency syndrome))*” [All Fields])) AND ((epidemiology) OR (prevalence) OR (Magnitude) OR (incidence)) AND ((associated factors) *” [All Fields])) OR (risk factor) OR (risk factors) OR (risk) OR (determinant factors) OR (determinants factors)) *” [All Fields])) AND (( Northern Ethiopia) OR (Southern Ethiopia) OR (Eastern Ethiopia) OR (Western Ethiopia) OR )Central Ethiopia)) OR (Ethiopia))*” [All Fields])) | 415 |
| Google Scholar | Suicidal ideation OR suicidal ideation OR suicide OR suicidal attempt OR suicidal behavior OR suicidal thought OR suicidal plan AND people OR individuals OR patients OR male OR female OR adults OR mothers AND HIV OR Human Immunodeficiency Virus OR AIDS OR acquired immunodeficiency syndrome AND epidemiology OR prevalence OR Magnitude OR incidence AND associated factors OR risk factors OR determinant AND Northern Ethiopia OR Southern Ethiopia OR Eastern Ethiopia OR Western Ethiopia OR Central Ethiopia OR Ethiopia | 4590 |
